# Research progress on post-translational modification of proteins and cardiovascular diseases

**DOI:** 10.1038/s41420-023-01560-5

**Published:** 2023-07-28

**Authors:** XueLi Cheng, Kai Wang, Yan Zhao, Kun Wang

**Affiliations:** 1 Key Laboratory of Birth Regulation and Control Technology of National Health Commission of China, Shandong Provincial Maternal and Child Health Care Hospital affiliated to Qingdao University, Jinan, 250014 Shandong China; 2 Institute for Translational Medicine, The Affiliated Hospital of Qingdao University, College of Medicine, Qingdao University, Qingdao, 266073 Shandong China

**Keywords:** Post-translational modifications, Cardiovascular diseases

## Abstract

Cardiovascular diseases (CVDs) such as atherosclerosis, myocardial remodeling, myocardial ischemia-reperfusion (I/R) injury, heart failure, and oxidative stress are among the greatest threats to human health worldwide. Cardiovascular pathogenesis has been studied for decades, and the influence of epigenetic changes on CVDs has been extensively studied. Post-translational modifications (PTMs), including phosphorylation, glycosylation, methylation, acetylation, ubiquitination, ubiquitin-like and nitrification, play important roles in the normal functioning of the cardiovascular system. Over the past decade, with the application of high-performance liquid chromatography-tandem mass spectrometry (HPLC-MS/MS), an increasing number novel acylation modifications have been discovered, including propionylation, crotonylation, butyrylation, succinylation, lactylation, and isonicotinylation. Each change in protein conformation has the potential to alter protein function and lead to CVDs, and this process is usually reversible. This article summarizes the mechanisms underlying several common PTMs involved in the occurrence and development of CVDs.

## Facts

- Post-translational modification is the last step in protein functional realization.
- Post-translational modification is a key mechanism to increase proteome diversity.
- Post-translational modification is important for the study of cardiovascular diseases.

## Open Questions

- What are the types of post-translational modification?
- Which cardiovascular diseases are associated with post-translational modification?
- How does post-translational modification affect cardiovascular disease?

## Introduction

Since the 21st century, cardiovascular disease (CVD) has been considered one of the most common diseases threatening human health worldwide. Owing to the progressive aging of the population, the prevalence and mortality of CVD are increasing annually, CVDs are threat to human health and have a significant impact on the quality of life and survival of patients. CVDs include heart and vascular diseases, such as atherosclerosis, myocardial remodeling, hypertension, dilated cardiomyopathy, myocardial ischemia-reperfusion (I/R) injury, heart failure, and oxidative stress. Although the symptoms of these diseases differ, the main mechanisms involved are related to signal transduction pathways and the mitochondrial oxygen supply capacity.

The occurrence and development of CVD are closely related to the environment and the behavior of patients. In recent years, epigenetic modifications have been shown to be involved in the pathophysiological process of CVDs. Epigenetic modification refers to reversible and heritable changes in gene function without any changes in the nuclear DNA sequence. These alterations include DNA methylation, histone modifications, histone acetylation, and RNA interference [1]. Post-translational modification (PTM) of proteins is the chemical modification of specific amino acid residues, which is ubiquitous in mammalian cells and plays a crucial role in the regulation of cellular molecular functions. Moreover, PTMs are reversible and functional regulators of eukaryotic cells. Currently, the commonly studied types of protein PTM include traditional acylation modifications, such as phosphorylation, glycosylation, methylation, acetylation, ubiquitination, ubiquitin-like, and nitrosylation. More recently identified acylation modifications, include propionylation, crotholylation, butylylation, succinylation, lactylation, and isonicotinylation. Some of these modifications have been shown to be involved in the regulation of signal transduction pathways, metabolism and other biological processes. Abnormal regulation of proteins after translation is a common feature of human diseases. For example, lysine methylation is a common PTM that affects many cellular pathways [2]; cellular Ca^2+^/calmodulin-dependent protein kinase II (CaMKII) phosphorylates cardiac ion channels at conserved serine/threonine residues, thereby affecting F-actin, which in turn reduces cardiomyocyte motility. Disorders involving altered CAMKII-dependent sarcoplasmic phosphorylation have been reported in patients with heart failure, resulting in changes in changes diastolic pressure [3]. Taking into account recent research advances nationally and abroad, this paper reviews the physiological and pathological effects of PTMs on CVDs.

## Types and biological functions of PTMs

PTMs are closely associated with the occurrence and development of CVDs. Traditional types of PTMs include phosphorylation, glycosylation, methylation, ubiquitination, and ubiquitin-like, which have been reported to modify various enzymes involved in CVD (Table 1). Modifications by new acylation methods include acetylation, propionylation, crotonylation, butylation, succinylation, lactation, and isonicotinylation. Acetylation is often classified as a traditional modification, whereas more recently identified acylation modifications are associated with signaling pathways, metabolism, and CVDs (Table 2). Here, we focused on the basic functions of common PTMs related to CVDs.

**Table 1:** Traditional PTM types, enzymes and their biological functions involved in CVD.

Modification			Modification sites	Biological functions	Cardiovascular diseases	References
Phosphorylation	PKs: (1) AGC kinases (PKA, PKG, PKC); (2) CaMK(CaMK,CDPK); (3) CMGC Kinases (CDKs, MAPKs, GSKs, CLKs); (4) PTKs; (5) Others (RLKs, CTRL)	PPs: PPP, PPM, PTP, Asp	Serine, Threonine Tyrosine	Signal transduction, growth and development, protein synthesis and metabolism, tumorigenesis, cell cycle	Atherosclerosis; Oxidative stress; I/R; Myocardial remodeling; heart failure	[94] [53] [95] [96]
Glycosylation	Glycosyltransferase,	Deglycation enzyme	Serine, Threonine, Hydroxyproline	protein folding and glycoprotein stability, protein sorting and packaging, cell communication, growth	I/R; Myocardial hypertrophy; heart failure	[97] [75] [98]
Methylation	PKMTs, PRMTs	PKDMs: LSD1, JHDM	Lysine, Arginine	RNA processing, Transcriptional regulation, signal transduction	Atherosclerosis; Oxidative stress; Myocardial hypertrophy	[99] [100] [101]
Ubiquitin	E1, E2, E3	DUBs: UCHs, USP/UBPs, OUTs, MJD, JAMM, MCPIPs, MINDYs	Lysine	apoptosis, signal transduction, DNA repairing, immunoreaction	Atherosclerosis; Oxidative stress; I/R; Myocardial remodeling, heart failure	[102] [103] [104] [105] [106]
Ubiquitin-like	E1, E2, E3	CSN5, NEDP1	Lysine	signal transduction, growth, immune response	Atherosclerosis; I/R; Myocardial remodeling, heart failure	[107] [108] [109] [110]

**Table 2:** Acylation mode, enzyme and biological function of CVD.

Modification	Writers	Erasers	Reader	Modification sites	Biological functions	Cardiovascular diseases	Reference
Acetylation	KAT1-17: (1) p300/CBP family (p300/CBP); (2) GNAT family (GCN5, PCAF); (3) MYST family (MOZ, MOF, Sas2, Sas3, Tip60); (4) SRC family (SRC-1,2, and 3); (5) Others (ACAT1).	HDAC family: (1) Class I (HDAC1,2,3); (2) Class II (IIa: HDAC 4,5,7 and 9: IIb: HDAC6 and 10); (3) Class III (SIRT1-7); (4) Class IV (HDAC11).	BRD3, BRD4, PBRM1, ENL, AF9	Lysine	Metabolism, cell cycle, signal transduction, stress	Atherosclerosis; I/R; Myocardial remodeling, heart failure	[111] [112] [113] [114]
Lactylation	p300, YiaC	HDAC1, HDAC2, HDAC3, CobB	_	Lysine	tumor development, inflammation, metabolic regulation	I/R; Acute heart failure and Myocardial hypertrophy(zebrafish)	[115] [93]
Crotonylation	p300/CBP, MOF, PCAF	HDAC1, HDAC2, HDAC3, HDAC8: SIRT1, SIRT2, SIRT3	Taf14, AF9, YEATS2, MOZ, DPF2	Lysine	Epigenetic inheritance, tumor metabolism, DNA damage repair	Myocardial hypertrophy	[116]
Succinylation	GCN5(KAT2A), HAT1, CPT1A, KGDHC,	SIRT3, SIRT5, SIRT7	GAS41	Lysine	inflammation, metabolic diseases, tumors	Atherosclerosis; I/R; heart failure	[117] [66] [118]
Isonicotinylation	p300/CBP	HDAC3	_	Lysine	cancer, tuberculosis	–	–

## Types of traditional modifications and their biological functions

### Phosphorylation

Protein phosphorylation is the most common, fundamental, and important regulatory mechanism regulating protein function and activity in living organisms. Protein phosphorylation refers to the transfer of the γ-phosphate group of ATP to one or more specific amino acid residues of the substrate protein, which is catalyzed by a protein kinase, or the regulation of GTP binding under the action of a signal. Protein phosphorylation occurs mainly at serine, threonine, and tyrosine residues. The main role of serine phosphorylation is to activate protein enzyme activity by alteration of the protein structure. Tyrosine phosphorylation has two functions: the first is the same as that of serine phosphorylation, and the second is to promote protein–protein interaction by binding to structural proteins, thereby promoting protein phosphorylation. The proportion of protein phosphorylation modifications among intracellular protein modifications is close to 1/3 [4].

Various protein kinases are involved in the phosphorylation of different amino acid residues. There are many types of protein kinases, two of which are mainly involved in the phosphorylation pathway: serine/threonine protein kinase and tyrosine protein kinases. Protein phosphorylation by protein kinase is a process, that is reversible by protein phosphatase-mediated dephosphorylation, thereby providing regulatory control of the activity of proteins and enzymes that respond to external signals.

**Fig. 1:**
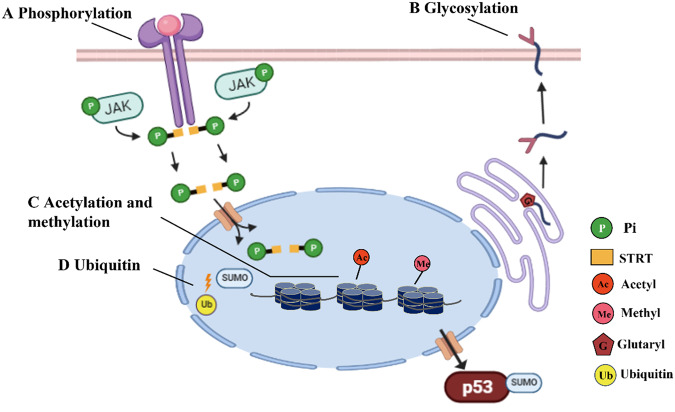
Traditional protein PTMs types. **A** Phosphorylation, this process is catalyzed by a protein Kinase, which transfers the gamma phosphate group of ATP or GTP to amino acid residues of the base protein, while the reverse process is removal of the corresponding phosphate group by protein phosphatase. **B** Glycosylation, Glycosylation is the process of adding sugars to proteins or lipids under the control of enzymes, starting from the endoplasmic reticulum and ending in the Golgi apparatus. Glycosyltransferase transfers sugars to proteins and forms glycosidic bonds with amino acid residues on the proteins. Proteins undergo glycosylation to form glycoproteins. **C** Acetylation and methylation, acylation is the process of transferring an acetyl group, such as acetyl-coA, to a protein lysine residue under the catalysis of acetyl transferase. Acetylation modification is regulated by acetyltransferases (HATs/KATs) and deacetylases (HDACs/KDACs). The sites of histone methylation are lysine and arginine. **D** Ubiquitination, Ubiquitination refers to a process in which one or more ubiquitin molecules (a polypeptide composed of 76 amino acids) classify proteins in cells under the action of a series of special enzymes, select target protein molecules from them, and carry out specific modification on the target protein. JAK Janus kinase, is a nonreceptor tyrosine protein kinase. STAT a signal transduction and transcription activating protein, is a unique family of proteins that can bind to DNA. P phosphate group. G Glycosyl. Ub ubiquitin. SUMO small ubiquitin like modifier. Ac acetyl. Me methyl.

Protein phosphorylation plays a role in almost every process of life, such as muscle contraction, cell signal transduction, cell growth and development, protein synthesis and metabolism, tumorigenesis, and learning and memory-related neural activity in higher animals. Baicalin (a Chinese herbal extract) has been shown to reverse myocardial damage and reduce inflammation after I/R via the JAK/STAT pathway, by inhibiting JAK2 and STAT3 phosphorylation levels [5]. Isoproterenol (ISO) -induced myocardial hypertrophy in rats is characterized by cardiac Janus kinase-mediated phosphorylation of signal transducers and transcriptional activators, resulting in significant upregulation of nuclear factor-κB. We have reported that simvastatin prevents the development of cardiac hypertrophy by modulating the JAK/STAT pathway in the heart of isoproterenol administered animals [6] (Fig. 1A). Glucagon-like peptide-1 and its analog exendin-4 (EX-4), exert cardiovascular protective effects. Activation of glucagon-like peptide-1 receptor induces phosphorylation Akt/endothelial nitric oxide synthase (p-Akt/p-eNOS), thereby upregulating the PI3K/Akt/eNOS signaling pathway [7]. The neuroprotective effects of PEX-4 in diabetic rats may be related to inhibition of the endoplasmic reticulum stress mediated by GP91 and CHOP, apoptosis mediated by Bax/Bcl-2/caspasE3/PARP, inflammation mediated by NF-κB/ICAM-1, and neurodegeneration induced by GFAP. This may be achieved via the p-Akt/p-eNOS-mediated signaling pathway. These results suggest possible therapeutic application of GLP-1R agonist PLGA microspheres in the treatment of neurological and cardiovascular complications in diabetes [8].

### Glycosylation

Protein glycosylation is also one of the most common PTMs that occur in the endoplasmic reticulum and refers to the covalent linking of oligosaccharides to protein amino acid residues to form glycosidic bonds under the control of glycosyltransferases (Fig. 1B). There is extensive protein glycosylation in eukaryotic cells, resulting in the generation of glycoproteins, and this PTM, plays an important role in regulating protein function [9].

Glycosylation in mammals is generally divided into three types according to the nature of the glycosidic bond: (1) O-linked glycosylation, carried out in the Golgi apparatus, whereby N-acetylgalactose is covalently connected to the oxygen of the hydroxyl groups of protein Ser, Thr, and Hyp residues to form glycoproteins. This connection mostly occurs in mucus and immunoglobulin proteins. (2) N-linked glycosylation, which starts in the endoplasmic reticulum and is completed in the Golgi apparatus, refers to the formation of a sugar chain by covalent connection with the free radical-NH2 of aspartic acid. This type of connection is often found in body fluids such as plasma, which is hence referred to as plasma glycoprotein; (3) GPI glycosyl phospholipid inositol anchor: C-terminal of GPI-anchored proteins are bridged to the core glycan by ethanolamine phosphate, and another phospholipid structure connects the GPI anchor to the cell membrane. Some receptors, differentiation antigens, and hydrolases have been confirmed to bind to the cell membrane through their GPI structure, which is the only way to connect them to the cell membrane.

There are four main functions of protein glycosylation: (1) glycosylated proteins, for which the oligosaccharide chains can promote protein folding and enhance glycoprotein stability; (2) facilitation of sorting and packaging in the Golgi apparatus and ensuring unidirectional transfer of glycoproteins from the rough endoplasmic reticulum to the Golgi membrane; (3) mediation of bidirectional communication between cells or participation in various processes such as differentiation and development; and (4) polyhydroxy sugar side chains an also affect the water solubility and charge of proteins. According to previous research, 70% of the total protein in the human body is post-translationally modified by glycosylation [10], and 1% of the human genome is involved in the synthesis and modification of glycoprotein [11].

Notch plays an important role in the development of animal embryonic nervous systems. Studies have shown that Notch1 is also involved in neuronal apoptosis, axonal retraction, and ischemic stroke in neurodegenerative diseases [12]. Glycosylation of the Notch receptors does not impair its function, but errors in the modification process cause secretase to cleave Notch, and this process may be implicated in the pathogenesis of Alzheimer’s disease [13]. Post-translational modification of cellular proteins by *O*-linked-beta-D-N-acetylglucosamine (*O*-GlcNAc) is involved in the signal transduction pathway related to the pathophysiology of CVD. This highly dynamic protein modification may induce functional changes in proteins and regulate key cellular processes, including translation, transcription, and cell death [14].

### Methylation

Protein methylation is catalyzed by methylases and acts on specific residues of proteins (Fig. 1C). Depending on the amino acids in the substrate, protein methyltransferases mainly include lysine methyltransferase, arginine methyltransferase, histidine methyltransferase and aspartic acid methyltransferase [15]. Protein methylation has been studied in both eukaryotes and prokaryotes. Methylation is an important dynamic modification and biological phenomenon involved in the regulation of proteins and protein pathways. Lysine methylation includes mono-, di- and trimethylation. Lysine methylation occurs by lysine methyltransferase-catalyzed transfer one to three methyl groups from S-adenosine adenosine methionine (SAM) to an ε-amine side chain of a lysine residue. Arginine methylation includes monomethyl and dimethylation [16]. Under the catalytic action of arginine methylation transferase, one or two methyl groups are added to a guanidine moiety to form a side chain that is mainly involved in RNA processing [17], transcriptional regulation [18], signal transduction [19].

### Ubiquitination

Ubiquitin is a highly conserved polypeptide composed of 76 amino acids that is widely present in eukaryotic cells. Protein ubiquitination is dependent on the ubiquitin–proteasome system for non-lysosomal protein degradation in the cytoplasm and nucleus. The classical function of ubiquitin is participation in a broad range of aspects of cellular functioning, including mitosis, proliferation, apoptosis, and intracellular signal transduction. The non-classical functions are closely related to cellular inflammation, DNA repair and metabolism, immune responses, intracellular localization, receptor regulation and other cellular processes underlying physiological activities. All these functions depend on the covalent connection of ubiquitin molecules to substrate proteins. The degradation of ubiquitin-proteins is a three-step enzymatic process. These enzymes are the ubiquitin-activating enzyme E1, the ubiquitin-binding enzyme E2, and the ubiquitin ligase E3. The process of ubiquitin activation is as follows: the ubiquitin monomer forms an intermolecular thioester bond with E1 under the action of adenosine triphosphate. The activated ubiquitin is then transferred to the cysteine residue of the active center of E2 to form the E2-ubiquitin complex, followed by E3 specifically recognizing and binding to the target protein and catalyzing the binding of ubiquitin to the amino group on the lysine residue of the target protein. Ubiquitin protein is degraded by the proteasome (Fig. 1D).

Protein ubiquitin modifications are closely related to cellular inflammation and immune responses. The nuclear factor-kappa B (NF-κB) family has been reported to be a key factor in regulating immune response and inflammatory response, and ubiquitin modification is essential in this process. PD-1/PD-L1 is a major pathway that regulates the immune escape checkpoint in cancer patients. Ubiquitination and de-ubiquitination of PD-1/PD-L1 play important roles in maintaining the stability and dynamic regulation of the PD-1/PD-L1 proteins. Therefore, PD-1/PD-L1 ubiquitination is a potential target for tumor immunotherapy [20]. Ubiquitinated K270 has been identified by mass spectrometry, confirming that the Arrdc4 adaptor of the Nedd4 family of ubiquitin ligases, plays an important role in extracellular vesicle biogenesis. It has also been shown that NEDD4-2 poly-ubiquitiation of K-29-linked Arrdc4 at K270 is a possible new signal for ARRDC4-mediated release of extracellular vesicles [21]. The N-methyl-D-aspartate (NMDA) glutamate receptor plays important roles in the development and storage of information in the mammalian brain. Interaction between NR1 and ubiquitin ligase Fbx2 is involved in the homeostatic control of NMDA receptors [22].

### Ubiquitin-like modifications

Two main types of ubiquitin-like modified proteins have been identified to date. One is Small Ubiquitin-like Modifier (SUMO), which has molecular weight of 11 kDa. SUMO mainly exists as five homologs in mammals. The binding mode of SUMO to substrate proteins is similar to that of ubiquitin modification, which is a highly dynamic and reversible process in vivo [23]. The other modification is Neddylation, which refers to the process of covalent binding of neural precursor cell expressed developmentally down-regulated 8 (NEDD8) protein and the target protein. A number of studies have shown that the binding mode of NEDD8 differs from that of ubiquitination. Because of differences in structure of the protein interactions mediated by ubiquitin and NEDD8, they are not interchangeable in cells. However, the binding mode is accomplished by an E1-E2-E3 multi-enzyme cascade reaction [24]. Ubiquitination is involved in regulation of the cell cycle, signal transduction, development, immune responses and many other important physiological activities.

## New types of acylation and their biological functions

### Acetylation

Protein acetylation refers to the addition of an acetyl is moiety added to a lysine residue of the substrate protein by an acetyltransferase, which is a mechanism by which cells control gene expression, protein activity, or physiological processes (Fig. 1C).

Protein acetylation occurs in both eukaryotes and prokaryotes. There were two types of acetylation. Protein N-Terminal protein acetylation is irreversible, whereas lysine acetylation is a reversible dynamic modification process [25]. The acetylation and deacetylation of proteins are dynamically balanced in the nucleus, precisely regulating the transcriptional expression of genes.

Protein acetylation not only plays a role in chromosome structure and activation of transcriptional regulators in the nucleus but also under physiological conditions, and a large number of non-nuclear proteins are acetylated. Acetylation is ubiquitous in cell metabolism, the cell cycle, cell signaling and cellular stress. It is highly specific and, has ample potential for the development of clinical drugs. Studies in animal models have shown that lysine acetylation plays a regulatory role in angiogenesis, hypertension, arrhythmia, heart failure, and vascular diseases. For example, lysine deacetylase inhibitors II and III have protective effects on the heart and vascular diseases, while class I inhibitors have protective effects on vascular injury but are harmful to the heart [26].

### Lactylation

**Fig. 2:**
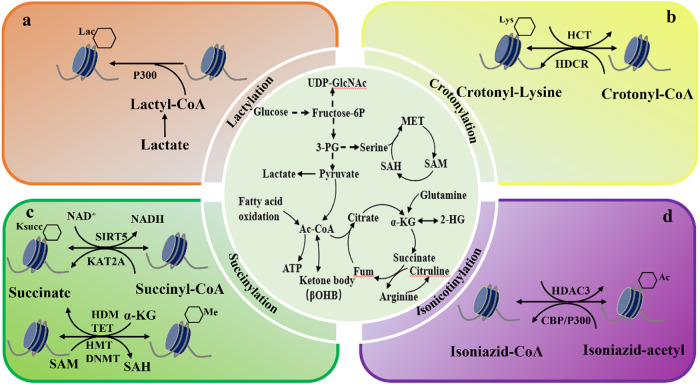
Relationship between novel acylation modification types and cell metabolism. Glucose, amino acids and other nutrients are metabolized by cells to produce acetyl-coA, NAD^+^, ATP and other metabolites, which participate in the protein PTMs. **a** Lactylation modification, in which lactate generates lactate coenzyme a and connects lactate group to histone lysine residue under the catalysis of p300. **b** Crotonylation, crotonylation modification refers to a modification produced by crotonyl transferase (HCT) transferring crotonyl to lysine residue using crotonyl coenzyme A (Cr-coA) as substrate. **c** Succinylation, the process by which a succinyl donor covalently binds a succinyl group to a lysine residue by enzymology, etc. **d** Isoniacylation, isoniazid and its metabolites induce PTM of histones-lysine isoniacylation (Kinic). Further studies showed that Kinic modification is dynamically regulated by CBP, P300(acetyltransferase) and HDAC3(deacetylase). Lac Lactate, Lys Lysine, HCT Crotonyl transferase, HDCR Histone decroton acylation, Ksucc, KAT2A, GNAT family of histone acetyltransferases, SIRT5 Desuccinylation catalytic enzyme, HDM, TET, Histone demethylase, HMT, DNMT, DNA methyltransferase, SAM S-adenosyl methionine, SAH S-Adenosylhomocysteine, HDAC histone deacetylase, CBP/P300 Histone acetyltransferase, Me methyl, Ac acetyl, Kbhb Histone lysine β-hydroxybutyrylation, Fum fumarate.

Lactate was first reported by Professor Yingming Zhao at the University of Chicago in 2019 [27] (Fig. 2a). When M1 macrophages are cultured under hypoxic conditions, incomplete oxidation of glucose leads to the accumulation of the metabolite lactic acid. Lactate modification of lysine refers to the production lactyl-CoA from lactic acid, followed by the lactyl to group becoming bound to the lysine sidechain under the action of acyltransferase. The Warburg effect refers to the altered energy metabolism in tumor cells. Even under aerobic conditions, glucose metabolism in tumors is more inclined toward glycolysis, resulting in increased lactic acid production. However, this accumulated lactic acid has long been regarded as a simple energy source for cells material and metabolic waste, and its important regulatory role in biological functions remains underappreciated. Recent studies have found that lactic acid accumulates during metabolism and can be used as a precursor or lactate modification of histone lysine residues by lactylation, thereby participating in homeostasis regulation of M1 macrophages infected by bacteria. Lactate can regulate gene expression through epigenetic modification of histones by lactylation [27]. However, the Warburg effect is characterized by the production of a large amount of lactic acid by aerobic glycolysis, suggesting that it may be involved in the occurrence of diseases through epigenetic inheritance. The discovery of histone lactate has linked cell metabolism with gene regulation, thus opening up a new research direction in cancer [28], immune responses [29], neurodegenerative diseases [30], and other pathologies, and it provides a new perspective for understanding the role of lactate in diseases.

In early 2020, the research group of James J. Galligan at the University of Colorado in the United States successively published evidence that proteins that can undergo lactylation can be involved in gene expression regulation through epigenetic modification of histone by lactatylation [31]. Zhao et al. research team digested human MCF-7 cells with trypsin and detected core histones using high-performance liquid chromatography-tandem mass spectrometry (HPLC-MS/MS). The found that the lysine residues of the three proteolytic peptides exhibited had mass shifts of 72. 021 Da, which corresponds to the mass shift caused by the addition of a lactate group to the ε-amino group of a lysine residue [27]. Extensive lactylation of histone lysine residues has been verified using isotope metabolism labeling techniques and a variety of in vivo and in vitro experiments. However, the two studies to date have different views on the properties of lactic acid modification, the source of the substrate, and the modification of the target protein. In one of these studies, the substrate of lactylation modification was lactate coenzyme A, which is an active enzymatic reaction. This study focused on lactylation of histones, and reported that lactylation is a novel epigenetic modification that directly stimulates gene transcription of chromatin [27]. However, according to the second study the substrate for lactylation is lactoylglutathione (LGSH), which is a passive non-enzymatic reaction. This study found evidence of lactylation of metabolic enzymes and reported that lactylation of related enzymes could result in negative feedback mediation of the glycolytic pathway [31]. Based on follow-up studies, protein lactylation modification has opened up a new field and provided new ideas on the potential mechanism of modification by lactic acid in the study of tumors, metabolism, immunity, and other physiological and pathological processes.

### Crotonylation

Crotonylation is a covalent modification catalyzed produced by crotonyl-transferase (HCT) using crotonyl-coenzyme A (Cr-coA) as a substrate to transfer the crotonyl group to the lysine residue (Fig. 2b). This an enzyme-dependent, dynamic equilibrium process. Croton acylation is a new type of acylation modification that was first discovered by Professor Yingming Zhao’s research team in 2011 [32]. Sixty-seven histone croton acylation sites have been identified. In somatic cells, histone croton acylation modification was enriched 200–300 upstream and downstream of the transcription start site, and the enrichment was symmetrically distributed at the center of the transcription start site [32]. These results indicate that modification of histone croton acylation is a marker for the precise initiation site of promoters and active genes, and it has been used as a specific marker for active chromosome-linked genes in human and post-meiotic mouse female germ cells. Presently, the specific metabolic pathways involved in crotonylation are unclear. In 2017, the research group of Professor Hongquan Zhang at Peking University proposed for the first time that crotonylation modification also occurs on non-histone proteins, and some overlapping sites of acetylation modification and crotonylation modification were found in the study, suggesting that the two modifications may be related [33]. Lysine crotonylation has been highly conserved during evolution between zebrafish and humans. Mass spectrometry analysis of zebrafish larvae revealed, extensive crotonylation of non-histone proteins, which has laid the foundation for future research on the effects of crotonylation on aging and heart failure [34].

### Succinylation

Lysine succinylation was first reported in 2011 by Yingming Zhao’s team at the University of Chicago, which found that there are many succinylation binding sites in *E. coli* [35]. Subsequent studies showed that succinylation, such as of histones, also occurs in animal tissues. Succinylation mainly occurs at lysine residues and is mediated by succinyl-CoA, which transfers a negatively charged four-carbon succinyl group to the primary amine of lysine residues (Fig. 2c). Compared with the classical modification method, the linking of amino acid residues with large-molecular-weight succinylates can lead to significant changes in the structure, physicochemical properties, and functions of many proteins. This is because the lysine group undergoing succinylation is conferred two negative charges, changing its valence from +1 to −1, and the succinylation results in a larger sidechain, so the structure and function of the protein undergo considerable change. Succinylation is a new acyl modification that involves carboxylic acid. Lysine succinylation has been shown to be a naturally occurring lysine modification [35]. Succinylation is involved in almost all biological processes in organisms. It is, therefore, relatively conserved throughout biological evolution. With the continuous development of mass spectrometry, amber acylation enzyme SIRT5 protein has been found to mainly exist in the mitochondria, regulating the amber acylating levels involved in mitochondrial metabolism, the Krebs cycle, metabolism of amino acids and fatty acid metabolism, and multiple metabolic pathways, with amber acylation also occurring in cytoplasmic and nuclear proteins [36]. It is closely related to the occurrence and development of cardiovascular diseases, tumors, inflammatory nervous system diseases, novel coronavirus disease, and liver-, lung-, and age-related diseases.

### Isonicotinylation

In 2021, the research group of Professor Hongquan Zhang at the School of Basic Medical Sciences of Peking University reported for the first time the modification by histone isonicotinylation [37]. It was found that isoniazid and its metabolites induced PTM of lysine. CBP/P300 was found to be a histone isonicyltransferase, and deacetylase HDAC3 was found to be a histone deisonicotinylase (Fig. 2d). Isonicotinylation is a dynamic regulatory process. Similar to other acylation modifications, isonicotinylation can promote gene transcription by loosening the chromatin structure in the genome, and it can induce histone to activate the PI3K/Akt/mTOR signaling pathway, thus providing a new perspective for the study of histone isonicotinylation and cancer [37]. The findings of this study add new information on protein post-translational modification and provide new possibilities for future studies.

## PTMs and CVD

**Fig. 3:**
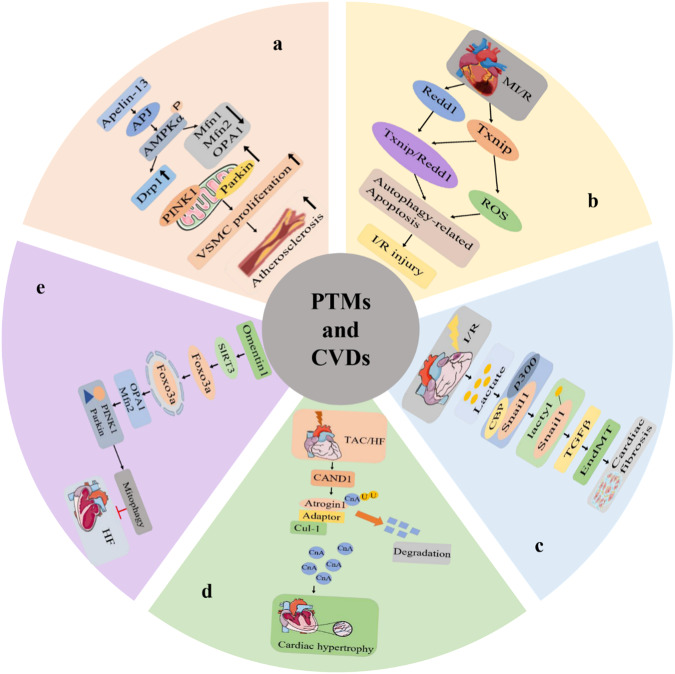
PTMs involve some mechanisms in CVDs. **a** Schematic diagram of PINK1/Parkin‐dependent mitophagy is responsible for apelin‐13‐induced human aortic VSMC proliferation and atherosclerotic lesions. **b** Schematic diagram of TXNIP-Redd1 expression is a novel signaling pathway that contributes to I/R injury by exaggerating excessive autophagy during reperfusion. **c** Schematic diagram of lactate-induced EndoMT after MI. **d** Schematic diagram depicting the proposed signaling mechanisms underlying the effects of CAND1 in the setting of cardiac hypertrophy. **e** The schematic of omentin1 ameliorated ischemia-induced HF via maintaining mitochondrial dynamical homeostasis and mitophagy. VSMC vascular smooth muscle cells, AS Atherosclerosis, PINK1 phosphatase and tensin homolog (PTEN)‐induced kinase 1, AMPK Adenosine monophosphate‐activated protein kinase, Drp1 dynamin‐related protein 1, Mfn1 and Mfn2 mitochondrial membranes mitofusin 1 and 2, OPA1 optic atrophy 1, Apelin a bioactive peptide and the ligand of the G protein-coupled receptor APJ, parkin E3 ubiquitin-protein ligase parkin, Omentin1 a novel adipokine, Foxo3a forkhead box O3a, CAND1 Cullin-associated and neddylation-dissociated 1 protein, EndoMT Endothelial-to-mesenchymal transition, TGF-β transforming growth factor–β, Snail a zinc finger transcription factor, Cul1 ubiquitin-protein ligase, CnA calcineurin, Atrogin-1 E3 ubiquitin ligases, Adaptor adaptor protein.

CVD, also known as circulatory disease, comprises several diseases involving the circulatory system, including myocardial remodeling, dilated cardiomyopathy, myocardial ischemia-reperfusion, atherosclerosis, heart failure, and oxidative stress. The main causes are disturbances in the oxidative energy supply of mitochondria, changes in signal transduction, and defects in cardiac diastolic and systolic blood pressure. PTMs are involved in the occurrence and development of various CVDs (Fig. 3). Here, we discuss research progress on PTMs in a series of CVDs.

### PTMs and atherosclerosis

Atherosclerosis is a chronic inflammatory cardiovascular disease that poses a significant threat to human health. In atherosclerotic lesions, modification by SUMO is involved in the regulation and localization of the transcriptional activity and stability of target proteins. Liver X receptor (LXR) is a cholesterol-sensing nuclear receptor and regulator of lipid metabolism that inhibits inflammatory signal transduction in macrophages through a trans-inhibition mechanism [38]. The expression of LXRs in macrophages can relieve atherosclerosis [39, 40]. Bi et al. found that LXR-mediated suppression of inflammatory genes is related to inhibition of the NF-κB pathway and SUMOylation: the binding of IκBα and SUMO2/3 promotes the degradation of IκBα, and LXR maintains the stability of IκBα by inhibiting SUMOylation, thus weakening the NF-κB signal in endothelial cells, which may play an anti-atherosclerotic role [41].

AMPKα1 has been found to directly phosphorylate the activated STAT-1 protein inhibitor (PIAS1) and promote the activity of Runx2-SUMO E3 ligase. Mutation of serine 510 and activated STAT-1 protein inhibitor inhibited metformin-induced Runx2-SUMO and blocked the effect of metformin on the reduction of Runx2 expression in new vascular smooth muscle cells triggered by oxidized low-density lipoprotein, thereby promoting atherosclerotic calcification of atherosclerosis [42]. The endothelial protein epsin interacts with ubiquitinylated endoplasmic reticulum protein inositol 1,5-triphosphate receptor 1 (IP3R1), thereby inducing proteasomal degradation of the calcium release channel [43]. Inhibition of the endothelial protein epsin prevents the development of atherosclerosis and inflammation. Thus, epsin-mediated IP3R1 degradation may serve as a new therapeutic target for the treatment of atherosclerosis and other diseases [44]. Casein kinase 2 (CK2) is a serine/protein kinase, inhibition of CK2 can prevent vascular smooth muscle cells from accumulating in the neointimal chamber and reduces the risk of accelerated atherosclerosis [45, 46].

### PTMs and oxidative stress

Oxidative stress is a process whereby the body is stimulated by external stimuli to produce excessive reactive oxygen radicals or reduce reactive oxygen scavengers, leading to cytotoxicity in most cells and eventually apoptosis. Several cardiac hypertrophy pathways are regulated by ROS. For example, angiotensin and endothelin act in a signaling cascade amplification by participating in ERK1/2, Akt, and NF-κB activation pathways, or the location of histone deacetylase [47–49]. At moderate concentrations ROS [50] play important roles in signal transduction under physiological conditions. However, excessive or sustained ROS production exceeds the existing antioxidant defense systems, resulting in oxidative stress [51]. Under physiological conditions, eNOS produces NO, which is an important vascular protective factor in endothelial cells. However, eNOS may become dysfunctional under oxidative stress-related pathological conditions [52].

Using a cardiac lipotoxic transgenic mouse model, it was found that lipid overload-induced mitochondrial redox reaction increased the ubiquitination of kinase anchor protein 121 (AKAP121), resulting in a decrease in DRP1Ser637 phosphorylation and alteration of the proteolysis process of OPA1, thus over-activating mitochondrial fission. Reduction or removal of mitochondrial ROS can restore mitochondrial morphology, both in vitro and in vivo [53]. Hydroxymethylglutaryl-CoA reductase degradation protein 1 (Hrd1) is an endoplasmic reticulum transmembrane E3 ubiquitin ligase. Hrd1 knockdown activates ER stress proteins, leading to cell death. When the knockout mice were placed in a state of adverse psychological stress, their cardiac function was found to be impaired. Overexpression of Hrd1 reduces pathological myocardial apoptosis, and cardiac hypertrophy in mice could be alleviated [54–56]. Protein nitrosylation is closely related to oxidative stress and occurs by an increase in reactive oxygen or nitrogen species under pathological conditions. It mainly refers to the process of protein tyrosine nitrosylation into 3-nitrotyrosine. Reduced serum ferric oxidase I (FeOxI) activity has been found to be associated with ceruloplasmin nitration and reduced survival in HF patients. Ceruloplasmin tyrosine nitration and cysteine thiol oxidation may play a role in the peroxynitrite-induced inhibition of FeOxI activity in vivo [57, 58]. Studies have shown that human and mouse SIRT5 protein expression is higher in heart tissues under stress conditions. Under stress conditions, SIRT5 knockout mouse hearts were subjected to ischemia-reperfusion injury, and the functional recovery and infarct size were evaluated. The kinetic energy recovery of SIRT5 knockout mice after stress treatment was found to be affected compared to the functional recovery of mice without stress treatment. Increased glycolysis may occur during I/R injury, resulting in increased lactate production. Studies have shown that inhibition of succinyl dehydrogenase can alleviate ischemia-reperfusion injury caused by SIRT5 depletion.

### PTMs and I/R injury

Acute myocardial infarction (AMI) is a severe hypoxic condition caused by coronary artery disease that results in myocardial necrosis. Currently, reperfusion is considered to be an effective treatment for acute myocardial infarction. However, recent studies have found that reperfusion can lead to further damage to the ischemic tissue. Some studies have found that I/R can cause calcium overload, leading to excessive mitochondrial division, reactive oxygen species explosion, and apoptosis of cardiac microvascular endothelial cells [59]. Histidine triad nucleotide-binding 2 (HINT2), combined with the mitochondrial calcium uniporter (MCU), inhibits ischemia–reperfusion injury and improves microvascular perfusion. The mechanism whereby microvascular perfusion is improved by overexpressing HINT2 may involve promotion of the expression and phosphorylation of endothelial nitric oxide synthase. Reperfusion injury occurs by inhibition of vascular endothelial growth factor-mediated cadherin phosphorylation [60–62]. Kelch ECH-associateded protein 1 (Keap1) is an E3 ubiquitin ligase. The cysteine residue of Keap1 forms a covalent bond with pubescenoside A (PBA). Furthermore, inhibition of nuclear factor erythroid 2-related factor 2 (Nrf2) ubiquitination and activation of antioxidant enzymes can improve myocardial ischemia-reperfusion associated with the NLRP3 inflammasome [63–65].

Myocardial infarction is a disease caused by a lack of energy; sirtuins are activated when energy is restricted, which can maintain cell metabolic homeostasis to reduce cell damage and prevent ischemic necrosis. SIRT5 is a mitochondrial NAD^+^-dependent deacetylase that promotes the removal of succinyl groups during deacetylation. The succinylation of SIRT5 is conserved; therefore, it is important to characterize this modification in detail this modification [66]. Using high-resolution mass spectrometry analysis, SIRT5 knockdown in the model of cardiac ischemia-reperfusion injury increases the infarct size, ultimately progressing to heart failure. Proteomic analysis revealed that the level of succinylation in SIRT5 knockout mice was significantly higher than that in wild-type mice. Pretreatment of SIRT5 knockout mouse hearts with succinate dehydrogenase competitive inhibitors reduced I/R injury, suggesting that alterations in succinate dehydrogenase activity have a definite effect on I/R injury and that succinate modification may play an important role in I/R injury [67]. I/R injury leads to metabolic disorders, and ischemia hypoxia leads to the production of a large amount of lactic acid in tissues, while activation of ion transporters increases intracellular sodium-calcium exchange, resulting in intracellular calcium overload. Lactate administration not only alleviates ischemic injury in animals, but also acts as a neuronal protector in adult humans and neonates [68].

### PTMs and myocardial remodeling

According to the pathological characteristics, myocardial remodeling mainly includes myocardial fibrosis and myocardial hypertrophy. Myocardial fibrosis, also known as myocardial calcification, refers to the deposition of fibrous connective tissue in the normal heart and myocardium, which leads to replacement of normal myocardial tissue with fibrotic collagen, resulting in myocardial ischemia and hypoxia [69]. Myocardial hypertrophy refers to the thickening of the myocardial wall that leads to narrowing of the heart cavity, which in turn leads to increased myocardial hypoxia, insufficient blood supply to the coronary arteries, and myocardial ischemia.

CK2 is a serine/protein kinase that is involved in novel human coronavirus disease, cancer, nervous system diseases, inflammation, autoimmune diseases, and cardiovascular diseases, such as cardiac ischemia-reperfusion injury, atherosclerosis, and myocardial hypertrophy [45]. The cell cycle regulator p27 (KIP1) has been reported to require CK2 for the treatment of cardiomyocyte hypertrophy [70, 71]. The catalytic subunit of CK2 destabilizes p27, which results in activation of autophagy and inhibition of apoptosis to protect the heart [72]. The protective effect of CK2 on cardiac hypertrophy is mediated by regulation of the phosphorylation of histone deacetylase 2 (HDAC2) [73]. Adenosine 5′-monophosphate -activated protein kinase (AMPK) maximizes AMPK activation to inhibit cardiac hypertrophy without affecting downstream targets. We demonstrated that AMPK inhibits O-GlcNAcylation by controlling the phosphorylation of GFAT, thus reducing O-GlcNAcylation such as of troponin. Therefore, AMPK activation prevents myocardial hypertrophy by inhibiting O-GlcNAcylation [74, 75]. Polycystic kidney disease 2-like 1 (PKD2L1, also known as TRPP3) is an acid sensor in taste cells. PKD2L1 knockout leads to myocardial hypertrophy and fibrosis, reduces cardiac mitochondrial phosphorylation, and promotes mitochondrial calcium overload. PKD2L1 deletion results in increased acetylation of histone 3 lysine 27 in the sodium/calcium exchange 1 (NCX1) promoter, which ultimately promotes cardiac hypertrophy [76–79].

WW domain-containing E3 Ub- protein ligase 1 (WWP1) is not only a therapeutic target for myocardial remodeling, but also a potential target for cardiac hypertrophy. WWP1 inhibits the signal transduction by regulating the polyubiquitin associated with Kmur27 to stabilize DVL2, thereby treating myocardial hypertrophy [80, 81]. The deacetylase sirtuin 3 (SIRT3), has been found to attenuate induced inflammation and profibrotic responses in human heart and neonatal rat cardiomyocytes [82]. Histone H3 lysine K27 acetylation on the DNA promoter promotes FOS transcription, cardiomyocyte fibrosis and inflammation through the FOS/AP-1 pathway, however, SIRT3 participates in histone H3DE deacetylation, which can inhibit the occurrence of cardiac hypertrophy [83, 84].

The source of reactive oxygen species in cardiovascular diseases is mainly NADPH oxidase (Noxes), which can activate non-receptor tyrosine kinases. The NOX family has seven members, of which NOX4 can bind to Src family non-receptor tyrosine kinases and phosphorylate NOX4 C-terminal tyrosine position residue 566. NOX4 activity in cardiomyocytes is negatively regulated to inhibit apoptosis after pressure overload and prevent cardiomyocyte remodeling [85]. Succinylation was primarily observed in the cytoplasm and mitochondria. GO enrichment analysis and KEGG pathway enrichment results showed that succinylated protein was mainly enriched in striated muscle hypertrophy and cardiac hypertrophy, and mainly involved in the oxidative phosphorylation pathway. Most studies to date have reported that succinylated proteins are mostly present in the mitochondria and are mainly involved in metabolic processes [86]. However, some studies have shown that succinylated proteins are located in the cytoplasm and may be involved in the regulation of hypertrophic cardiomyopathy. In hypertrophic cardiomyopathy, histone crotonylation is associated with cardiac hypertrophy, development, and muscle contraction [87].

### PTMs and heart failure

The expression of extracellular vesicle-related molecule miR-146a is increased in cardiomyocyte failure, and the activation of the SUMO1/SERCA2a signal axis affects cardiomyocyte function. Therefore, miR-146a is a new regulatory factor for targeted treatment of heart failure that regulates SUMOylation [88]. Cardiac Drp1 protein has been reported to be highly expressed and Drp1 Ser616 phosphorylation increased in mice fed a high-fat diet for 18 weeks. In adult cardiomyocytes, both palmitate and high-fat diets increased the acetylation of Drp1 at lysine 642 in mouse hearts, which is important for mitochondrial fission and cardiomyocyte death [89]. Both animal and clinical studies have shown that the the O-GlcNAcetylation level in heart tissue increases during heart failure, and fatty acid oxidation decreases in patients with heart failure, resulting in an increase in glucose entering the hexosamine biosynthesis pathway and an increase in the production of uridine diphosphate-GlcNAc (UDP-GlcNAc) under the action of rate-limiting enzymes, thereby resulting in an increase in the level of O-GlcNAcetylation. Continuous glycosylation can lead to heart failure and myocardial hypertrophy caused by cardiac metabolism [90, 91].

In heart failure, the major AMPKα isoform changes from AMPKα2 to AMPKα1. Phenylephedrine stimulates the activity of AMPKα2 and specifically interacts with the Ser495 site of phosphorylated PINK1. Phosphorylated PINK1 binds to E3 ubiquitin ligase to depolarize mitochondria, participates in cardiac mitochondrial autophagy, and accelerates heart failure [22, 64, 92]. Lactic acid derived from this herb inhibited inflammation and myocardial hypertrophy in zebrafish models of acute heart failure. This suggests that lactic acid is a protective agent for the heart and a novel therapeutic agent for acute heart failure [93].

## Conclusion

This review focuses on several common protein translational modifications and their effect on various CVDs. PTMs are ubiquitous in the occurrence and development of CVD, although the pathogenesis and pathological role are still unclear. Further studies on the mechanism of action PTMs on CVDs can broaden our understanding and improve treatment. As CVDs have serious impacts on people’s lives, they are a priority research focus.

In recent years, PTMs have gradually become a new hotspot in the field of medical research, as they are closely related to the occurrence of CVD. Currently, more than 450 unique modes of PTM have been identified, and this study is focused on the most common types. Most PTMs are reversible and control the state of the body by controlling the state of the cell. These types of PTM can not only control in protection from physiological insults, but also act concomitantly in several ways to ensure that cells can respond quickly and accurately to external stimuli. Unlike transcriptional translation, protein translation is a dynamic process that rapidly participates, which can quickly participate in barrier maintenance. PTMs occur mainly through participation in cardiovascular signaling pathways, mitochondrial oxidative stress, and cardiomyocyte apoptosis. It is well known that the incidence of CVD is high and detrimental, and the prevalence rate increases with increasing age. Therefore, finding new strategies for treating CVDs. We anticipate that continuous research in this field will improve the quality of life for patients with CVD.
